# Effectiveness of increasing the scalp cooling duration to prevent alopecia during adjuvant chemotherapy for breast cancer: a randomized pilot study

**DOI:** 10.1007/s00520-024-08579-z

**Published:** 2024-06-05

**Authors:** Edith Carton, Anne Mercier Blas, Clément Perret, Marcelle Le Bihan

**Affiliations:** 1 CHP Saint Grégoire, ICRB, Oncologie-Radiothérapie - boulevard de la Boutière, 35760 Saint Grégoire, France; 2 Direction des Soins Territoire Bretagne, Vivalto Santé, 9 boulevard de la Boutière, 35760 Saint Grégoire, France

**Keywords:** Alopecia, Breast cancer, Chemotherapy, Side-effects, Hair preservation, Scalp cooling

## Abstract

**Purpose:**

Alopecia is a common side-effect of chemotherapy and can be extremely distressing to patients. Scalp cooling can be used to reduce hair loss, but the optimal duration of cooling remains unclear. Our aim was to determine whether increasing the duration of scalp cooling improves hair preservation.

**Methods:**

Patients with HER2-negative, non-metastatic, breast cancer received scalp cooling during adjuvant chemotherapy: three cycles of epirubicin/cyclophosphamide (EC) followed by three cycles of paclitaxel. The patients were randomly assigned to two groups. Group A (n=18) wore a Paxman cooling cap during each infusion and for 30 min post-infusion while Group B (n=19) wore the cap from 30 min before to 2 h after each infusion. All patients were asked to complete a questionnaire recording hair loss/regrowth, adverse events, and quality of life. Success of treatment was defined as <50% hair loss.

**Results:**

The success rates after each of the three cycles did not differ significantly between the two groups (EC: Group A: 40%, Group B: 44%; paclitaxel: Group A: 50%, Group B: 36%; p>0.05). Hair regrowth was significantly higher in Group B at the 8-week follow-up, but not at the 6-month follow-up. Head discomfort affected more patients in Group B than in Group A during the first session (94% vs. 62%, respectively; p=0.039).

**Conclusion:**

Long duration scalp cooling during chemotherapy might increase patients’ discomfort and does not appear to improve hair preservation.

**Supplementary information:**

The online version contains supplementary material available at 10.1007/s00520-024-08579-z.

## Introduction

Breast cancer (BC) is the most commonly diagnosed malignancy in the world, with over 2 million new cases each year [1]. Treatment of BC has improved significantly and 5-year survival rates are currently around 85% for regional stage BC [2, 3]. Many patients with BC receive chemotherapy, working by inhibiting the division of rapidly dividing cancer cells. However, these drugs can also disrupt other rapidly proliferating cells (in the bone marrow, gastrointestinal tract, and hair follicles), leading to various side-effects including myelosuppression, mucositis, and alopecia.

Chemotherapy-induced alopecia can be severe as around 90% of hair follicles are diving rapidly at any time, making them susceptible to chemotherapy-induced damage [4]. Hair loss typically occurs within days to weeks after starting chemotherapy and usually persists until 1–3 months after the end of the treatment. The hair then begins to grow back again, often with a different color or texture [4], but for some patients the hair loss can be permanent [5].

Alopecia can cause profound psychological distress [6–9] and around 8% of patients consider refusing chemotherapy to avoid losing their hair [9]. Many studies have reported that the distress caused by alopecia may even exceed that of losing a breast [7, 10–12].

Different chemotherapeutic agents are more or less likely to induce alopecia. Topoisomerase inhibitors (e.g., epirubicin) lead to alopecia in 60–100% of patients and anti-microtubule agents (e.g., paclitaxel) induce alopecia in around 80% of patients [13]. In contrast, methotrexate, a DNA synthesis inhibitor, is unlikely to cause alopecia [14]. The incidence of hair loss is also dose-dependent and is higher when patients are administered several chemotherapeutic agents simultaneously [13]. In addition, permanent alopecia is more likely with certain chemotherapeutic agents, such as docetaxel, where it affects around 23% of patients [5].

Various strategies have been adopted to prevent chemotherapy-induced alopecia. At present, the most effective approach involves scalp cooling [15], when patients wear a close-fitting cooling cap containing a coolant that is continuously circulated through a refrigeration unit to maintain a constant low temperature [14]. The rationale is that the low temperature induces vasoconstriction, which reduces the amount of blood and drug reaching the scalp; in addition, cellular metabolism is slowed, rendering the cells less vulnerable to chemotherapeutic agents [16]. A recent meta-analysis (referring to several chemotherapy drugs, especially anthracyclines (epirubicin or doxorubicin), cyclophosphamide and taxanes – docetaxel or paclitaxel) showed that patients who received scalp cooling during chemotherapy had a 41% lower risk of alopecia compared to those who had no scalp cooling [17].

Although scalp cooling is increasingly offered to patients, there are currently no clear guidelines on how to optimize the effectiveness [18]. For drugs with a long elimination half-life, the cooling cap is often kept on for several hours after the end of each infusion [19, 20], whereas for other chemotherapeutic agents such as docetaxel, reducing the post-infusion cooling time may have a beneficial effect on hair preservation [21, 22]. It has been suggested that shorter post-infusion cooling may lead to drug remains being ‘flushed out’ of the scalp more rapidly, thus protecting the hair follicles [14]. Contrasting results were found in patients administered fluorouracil, epirubicin, and cyclophosphamide where more moderate/complete hair loss was found for post-infusion cooling of 90 min as opposed to 150 min [23].

Our aim was to determine whether scalp cooling for longer durations is more effective at reducing alopecia in patients undergoing adjuvant chemotherapy (epirubicin + cyclophosphamide followed by paclitaxel) for BC. The secondary aims were to evaluate the tolerability of scalp cooling for longer durations and its effects on the patients’ quality of life (QoL).

## Methods

### Study design

This was an open label, randomized, prospective, monocentric, parallel-group, interventional study.

The study was conducted in accordance with the Declaration of Helsinki, the principles of Good Clinical Practice, and French law. Ethical approval was obtained from the Committee for the Protection of Persons (CPP Ouest V; registration number: 19/016-2). The study was registered with the French Agency for the Safety of Health Products (ANSM; 2018-A02259-46) and declared to the French National Commission on Informatics and Liberty (CNIL; 2204516). The CONSORT checklist was used during the writing of this paper [24].

### Study population

Included patients were women (sex defined at birth), aged ≥18-years, with histologically confirmed HER2-negative, non-metastatic adenocarcinoma of the breast. Women were included if they were about to start adjuvant chemotherapy consisting of three cycles of epirubicin (100 mg/m^2^) + cyclophosphamide (600 mg/m^2^) (EC), administered on the first day every 3 weeks, followed by three cycles of paclitaxel (90 mg/m^2^), administered on days 1, 8, and 15 every 3 weeks. Patients were only included if they were covered by the French social security, provided informed consent, and were treated and followed up at the Saint-Grégoire hospital (Brittany, France) for the total study duration (12 months).

Patients were excluded if they had: diseases affecting the scalp or hair which contraindicated the use of scalp cooling; metastatic adenocarcinoma of the breast; previous treatments that can cause hair loss; were participating in another clinical drug trial; Raynaud’s disease; cold agglutinin disease; cryoglobulinemia; or cryofibrinogenemia. Patients were also excluded if they were unable to be followed up for psychological, geographic, or other reasons, if they were deprived of liberty or under guardianship, or if they had another disease incompatible with study inclusion.

### Interventions

All patients received regular chemotherapy sessions over a period of 6 months, as indicated above. For each session, the patients wore a Paxman cooling cap (Paxman Orbis II System, Houston TX, USA), which contains a coolant that is circulated through a refrigeration unit to maintain a constant, low temperature. The cap is made of soft silicone and is designed to provide a close fit to the patient’s scalp. The device meets the IEC 60601:2006 safety standards for medical devices. The cap was placed on each patient’s head by the nurse (MLB).

The patients were randomly assigned to one of two groups: (1) group A, who wore a cooling cap from the start of each chemotherapy infusion to 30 min after the end; (2) group B, who wore a cooling cap from 30 min before the start of each chemotherapy infusion to 2 h after the end. Randomization was carried out using a computer program in Excel. The infusion took 30–40 min for EC and around 1 h for paclitaxel. The follow-up period was 6 months after the last chemotherapy session.

### Outcomes

The outcome measures were obtained using questionnaires (Online Resource 1) that were completed by the patients and the nurse.

#### Primary outcome measure

The primary outcome measure was the degree of hair loss, as rated by the nurse. The questionnaires were administered at the end of each of the three treatment cycles (EC and paclitaxel), and at the 8-week and 6-month follow-ups. The nurse rated the degree of alopecia as absent (grade 0), <50% (grade 1), >50% (grade 2), total (grade 3), or persistent (grade 4). Scalp cooling was considered successful if there was <50% hair loss [25].

#### Secondary outcome measures

The secondary outcomes measures included: (i) the rating of hair loss by the patient, as absent, partial or total, and “partial” was considered to be less than 50% hair loss; scalp cooling was also considered successful if there was <50% hair loss; (ii) whether the patient wore a head covering (e.g., wig, headscarf), as recorded by the nurse and the patient on the questionnaire; (iii) the quality of hair regrowth, as rated by the patient on a scale of 0–10 (0: no regrowth; 10: hair regrowth as it was prior to chemotherapy); (iv) tolerability/satisfaction, as assessed using the questionnaire at the end of each chemotherapy session when the cooling cap was removed. The questionnaire included items about side-effects, such as headaches, neck pains, and nausea. There was also an overall tolerability rating on a scale of 0–10. The questionnaires were completed by the patients; (v) QoL, as evaluated at inclusion, after each of the three chemotherapy cycles (EC and paclitaxel), and at the 8-week and 6-month follow-ups. This was assessed using the European Organization for Research and Treatment of Cancer QoL Questionnaire (EORTC QLQ-C30) Version 3 with the BR-23 supplement for BC [26]. Patients have to rate various items such as the difficulty walking long distances, the need for personal care, feeling weak.

Demographic characteristics and the characteristics of the patients’ hair prior to chemotherapy (e.g., color, density, use of hair dyes) were recorded at inclusion. These items were indicated by the patient.

### Sample size

Our study is a pilot study about effectiveness of longer duration of scalp cooling during chemotherapy.

The null hypothesis was that there is no difference in terms of hair preservation between prolonged scalp cooling between chemotherapy and short scalp cooling. For this pilot study, 12 participants were first enrolled in each group, giving a total of 24 patients. It was planned in the protocol to replace patients lost to follow-up or stopping the study with new patients, this is why thirty-seven patients were finally included, considering the importance of discontinued interventions. This point was approved by a national research ethic committee (Comittee for the protection of persons, CPP Ouest V).

### Statistical methods

Statistical analyses were carried out using EasyMedStat software (Levallois-Perret, France) with a significance level of p<0.05. Quantitative variables are summarized as the mean ± standard deviation (SD), and qualitative variables as frequencies and percentages. Significant differences between the two groups were determined using the Student’s t-test or Fisher’s test.

## Results

### Study participants

**Fig. 1:**
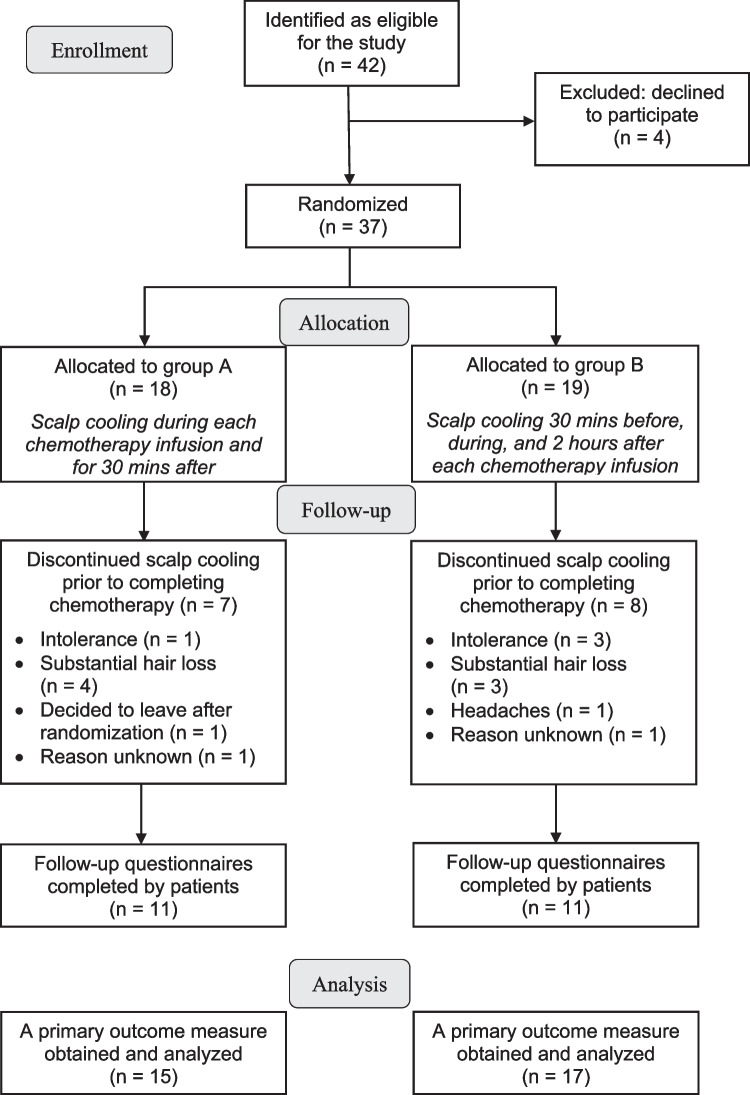
Flow diagram of the study participants

A total of 37 patients were recruited between November 18, 2019, and December 2, 2021; group A (n=18), group B (n=19) (Fig. 1). The characteristics of the participants are shown in Table 1. Of note, the patients were significantly older in group A than in group B (p=0.022).

**Table 1:** Characteristics of the Patients in Group A and Group B

	Group A	Group B	Difference (*p* value)
Age, years; mean ± SD	58.8 ± 10.7 (n = 18)	50.46 ± 10.21 (n = 19)	.022
Follow-up duration, months; mean ± SD	11.8 ± 0.4	11.3 ± 0.2	.09
3 cycles of EC with cooling cap; N (%)	15 (83.3)	17 (94.4)	
3 cycles of paclitaxel with cooling cap; N (%)	11(55.6)	11 (61.1)	
Number of exposures to cooling cap; mean ± SD	5.4 ± 2.3	5.1 ± 2.4	.75

Abbreviations: *EC*, epirubicin + cyclophosphamide; *SD*, standard deviation

Hair color was brown/black hair (n=22), blond (n=3), white (n=4), and chestnut/auburn (n=6); 25 women dyed their hair and nine used a hair dye containing ammonium. Prior to treatment, only one patient reported that their hair density was sparse (group B) and none reported any problems with their eyebrows, eyelashes, or body hair.

Some patients dropped out the study before the end; reasons are presented in Fig. 1. Most important reason was substantial hair loss, and intolerance to the cooling cap for some patients. Finally, follow-up questionnaires were completed by 11 patients in each group, and analysis of outcomes were done for 15 and 17 patients in groups A and B, respectively.

### Primary outcome measure

**Fig. 2:**
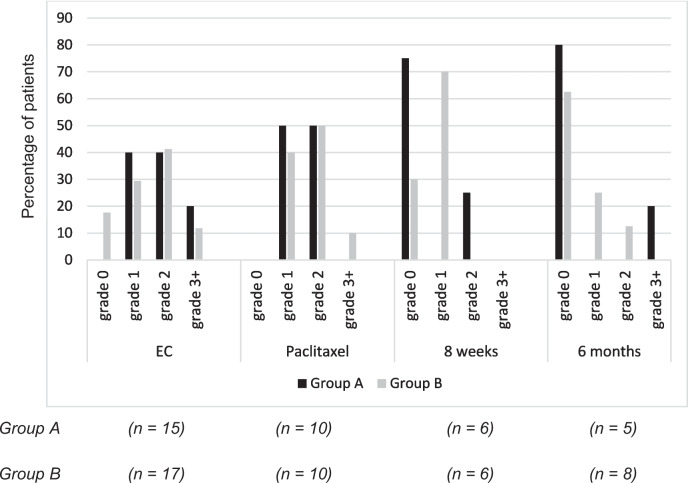
**Nurse’s ratings of the patients’ hair loss.** The percentage of patients with each grade of alopecia, as determined by a nurse after three cycles of epirubicin + cyclophosphamide (EC), three cycles of paclitaxel, and at the 8-week and 6-month follow-ups. Grade 0 = no hair loss, grade 1 = <50% hair loss, grade 2 = >50% hair loss, and grade 3+ = total hair loss

The hair loss rating by the nurse is shown in Fig. 2.

All patients were considered to have some degree of hair loss (grade 1+) following the EC treatment cycles, except for three patients, all in group B (17.6%). Three patients in group A (20%; aged 40-, 48-, and 71-years at inclusion) and two in group B (11.7%; aged 59- and 62-years at inclusion) were reported to have total hair loss (grade 3+); none of these patients continued with scalp cooling.

Following the paclitaxel treatment cycles, all patients were considered to have hair loss (grade 1+), but there were no patients with total hair loss in group A and only one patient in group B (10%). At the follow-up appointments, most patients were considered to have no hair loss (grade 0), indicating that their hair had regrown. Only one patient (12.5%; group A; 6-month follow-up) was considered to have total hair loss (grade 3+).

For the nurse’s ratings, the data were grouped into two categories: <50% hair loss (indicating treatment success) and >50% hair loss, as in previous studies [25]. This showed that scalp cooling was successful for 40% of the patients in group A and 47% in group B following the EC cycles, and for 50% of the patients in group A and 40% in group B following the paclitaxel cycles. There were no significant group differences between the groups following the EC cycles (p=0.41) or the paclitaxel cycles (p=0.67). Furthermore, there were no significant group differences at the 8-week (p=0.51) or 6-month follow-ups (p=0.75). These results indicate that the success of scalp cooling was not affected significantly by the cooling time.

### Secondary outcome measures

#### Hair loss rating by patients

**Fig. 3:**
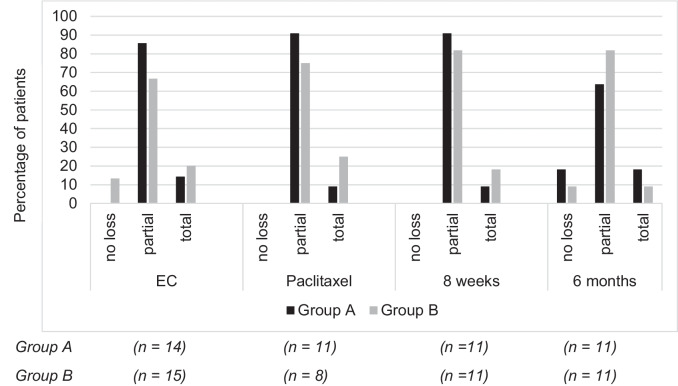
**Patients’ ratings of their hair loss.** The percentage of patients with each self-rated hair loss after three cycles of epirubicin + cyclophosphamide (EC), three cycles of paclitaxel, and at the 8-week and 6-month follow-ups

The patients’ ratings of their hair loss are shown in Fig. 3. Following the EC and paclitaxel treatment cycles, the patients largely reported having partial hair loss. The few patients who reported no hair loss were all in group B (EC: n=2). A small number of patients in both groups reported total hair loss after the EC and paclitaxel cycles (group A: EC, n=2, paclitaxel, n=1; group B: EC, n=3, paclitaxel, n=2). At the follow-up appointments, the patients mostly reported having partial hair loss. At the 6-month follow-up, three patients reported having no hair loss (group A: n=2; group B: n=1) and three reported having total hair loss (group A: n=2; group B: n=1).

A comparison of the two categories, no/partial hair loss vs. total hair loss, showed no significant differences between the two groups at any time point (EC: p=0.66; paclitaxel: p=0.36; 8-week follow-up: p=0.59; 6-month follow-up: p=0.59). These results indicate that the different scalp cooling times did not significantly alter the treatment outcomes.

#### Wearing a head covering

At the end of each treatment cycle, more than half of the patients in both groups reported wearing a head covering (Online Resource 2).

Due to the drop out rate during Paclitaxel cycles, follow-up analysis was not possible at the end of the total chemotherapy treatment, so analysis was limited to the patients’responses at the end of the EC cycles. At the follow-up appointments, the nurse recorded that few patients wore a head covering, while most patients reported that they wore one. This difference is probably because the patients recorded whether they ever wore a head covering whereas the nurse presumably noted whether the patients were wearing one during their visit. The nurse’s responses are in line with patient’s responses concerning hair regrowth in most patients. There was no significant difference between the two groups (p=0.68).

#### Quality of hair regrowth

The mean ratings for hair regrowth at the 8-week and 6-month follow-ups are shown in Online Resource 3. A comparison between the two groups showed a significant difference at the 8-week follow-up, with group B having higher regrowth ratings (p=0.015). The ratings at the 6-month follow-up were not significantly different (p=0.535).

A comparison between the two groups showed a significant difference at the 8-week follow-up, with group B having higher regrowth ratings (p=0.015). The ratings at the 6-month follow-up were not significantly different (p=0.535).

### Tolerability and adverse events

The results are shown in Table 2. Overall tolerability was similar in the two groups, with overall mean ratings of 4.5 (scale 0–10). More than half of the patients experienced head discomfort. This affected significantly more patients in group B than group A at the end of the first treatment session (94.1% vs. 62.5%, p=0.039), but this difference was no longer significant by the third session (69.2% vs. 91.7%, p=0.32). Around one-third of the patients also reported headaches, but there was no significant difference between groups.

**Table 2:** Results from the Tolerability Questionnaire

		Group A	Group B	*P* value (t-test / Fisher’s)
Overall tolerability, mean ± SD	*All sessions*	4.57 ± 2.48 (n = 17)	4.62 ± 2.64 (n = 18)	.96
Head discomfort, % of patients	*Session 1*	62.5 (10/16)	94.1 (16/17)	.039
	*Session 3*	91.7 (11/12)	69.2 (9/13)	.32
Corporal discomfort, % of patients	*Session 1*	13.3 (2/15)	17.6(3/17)	1.0
	*Session 3*	25 (3/12)	38.46 (5/13)	.67
Headache, % of patients	*Session 1*	31.25 (5/16)	35.3 (6/17)	1.0
	*Session 3*	41.7 (5/12)	38.5 (5/13)	1.0
Neck pains, % of patients	*Session 1*	6.25 (1/16)	11.8 (2/17)	1.0
	*Session 3*	25 (3/12)	23.1 (3/13)	1.0
Nausea, % of patients	*Session 1*	6.7 (1/15)	5.9 (1/17)	1.0
	*Session 3*	16.7 (2/12)	46.1 (6/13)	.20
Aches, % of patients	*Session 1*	0 (0/15)	11.8 (2/17)	.49
	*Session 3*	16.7 (2/12)	7.7 (1/13)	.59
Flu-like symptoms, % of patients	*Session 1*	6.7 (1/15)	0 (0/17)	.47
	*Session 3*	8.3 (1/12)	0 (0/13)	.48

Abbreviation: *SD*, standard deviation

#### Quality of life

Changes in QoL were determined using the scores prior to starting chemotherapy as the baseline (online resource 4**)**. Mean baseline score was 69.7 ± 28.0 for group A (n=13) vs. 78.2 ± 17.5 for group B (n=13) (t-test, p=0.61). A comparison of the changes in QoL between the two groups showed no significant difference at any time point (p>0.05). Thus, different scalp cooling times appeared to have no effect on QoL.

## Discussion

This study indicates that prolonging the duration of scalp cooling might not improve hair preservation in patients receiving EC or paclitaxel for BC. Specifically, wearing a cooling cap from 30 min before the infusion to 2 h after the end did not give significantly better results than wearing the cap during the infusion and for 30 min afterwards.

Although few patients in this study were considered to have no hair loss at the end of chemotherapy, a substantial proportion had <50% hair loss, indicating scalp cooling success [25]. This concerned 40% of patients in group A and 47% in group B following the EC cycles, and 50% in group A and 40% in group B following the paclitaxel cycles. These success rates are similar to those found during infusions of taxane/anthracycline [27, 28], with a success rate of 50.5% [29]. It should be noted that our success rates are markedly better than when there is no scalp cooling at all [29, 30]. However, these results should be interpreted with caution, due to the small size of our cohort.

Several studies have reported higher success rates than ours when patients received scalp cooling during chemotherapy. Rugo et al. reported that 66% of their patients had <50% hair loss [31]. However, these patients were administered non-anthracycline-based chemotherapy. Differences in success rates may also be drug dose-related. In a previous study, 52% of patients did not require a head covering when epirubicin was administered at 90 mg/m^2^ compared to 33% of patients when it was administered at 100 mg/m^2^ [32]. In our study, patients were also administered epirubicin at a dose of 100 mg/m^2^ and <50% of the patients did not wear a head covering following the EC cycles (29% according to the patients’ responses). Various other factors can also affect the success of scale cooling such as scalp temperature and hair type [32–34].

Our results also show that patients in group A who had scalp cooling for a shorter duration reported poorer quality hair regrowth at the 8-week follow-up. This suggests that scalp cooling for a longer duration may promote better quality hair regrowth. However, this benefit appeared to be short-lived as no significant group difference was found between the two groups at the 6-month follow-up (the mean rating for group A was still slightly lower). These findings should be interpreted with caution because they are not reflected by the hair loss ratings or the proportion of patients who wore a head covering. In addition, the differences may relate to the patients’ age since group A patients were significantly older than group B patients (59- vs. 50-years), and it has been shown that hair regrowth occurs earlier in younger, premenopausal women [35].

Low-grade side-effects may occur after scalp cooling [26, 36] and it is possible that scalp cooling for longer durations may increase the incidence and severity of these side-effects. Our results show a significant difference in the incidence of head discomfort at the first scalp cooling session, with 94% of patients affected in group B vs. 63% in group A. However, only four patients discontinued scalp cooling due to intolerance: 3 (group B) vs. 1 (group A). It is possible that some patients may become habituated to the cold after repeated exposure as the incidence of head discomfort was lower in group B at the third session (69%); no decrease was observed in group A. The incidence of other side-effects was generally low with no significant differences between the two groups. Headaches were the most common side-effect at the first session (31% in group A vs. 35% in group B), an incidence similar to previous studies [28, 37]. However, a longer duration of cooling did not result in a higher incidence of headaches.

As scalp cooling can reduce alopecia, it could also be expected to reduce distress and improve QoL. However, we found no significant differences between the changes in EORTC-QLQ-30 scores in the two groups. Thus, scalp cooling duration may not affect the patients’ QoL. A previous study showed that patients have a better QoL when scalp cooling is effective, but that QoL may worsen when the intervention does not live up to expectations [32]. Scalp cooling was effective (<50% hair loss) in almost half of our patients and this seemed mirrored by the QoL scores neither improving nor worsening. It is important to ensure that patients’ expectations are not too high prior to scalp cooling, as this can lead to distress when they are not met [9]. Furthermore, it is advisable not to offer scalp cooling when success is unlikely [32].

One of the limitations of this study is that the main outcome measures were subjective and therefore subject to bias, with potential differences between the patients’ and the nurse’s ratings. For example, the nurse mainly recorded that there was no hair loss at the follow-up appointments, particularly for group A, whereas the patients mainly reported partial hair loss at these time points. The patients may have overestimated the amount of hair that they had prior to chemotherapy, whereas the nurse may have been influenced by having seen early hair regrowth in other patients and so have judged it to be ‘normal/no hair loss’. Although 37 patients were recruited, only 11 patients in group A and 11 patients in group B completed the follow-up questionnaires, limitating the statistical power and increasing the risk of a type II error. A related limitation is that some patients dropped out of the study because of hair loss. This could distort the results at later time points, as these would mainly include patients with less extreme initial hair loss and so not reflect the entire group of patients. A further limitation is that we did not consider various factors that have been shown to influence the effectiveness of scalp cooling, including scalp temperature, variable between individuals receiving the same cooling treatment [18, 33, 37], , and menopausal status, where scalp cooling may be more effective in premenopausal women [34]. Finally, the study was limited to scalp cooling during EC and paclitaxel chemotherapy for BC in women. The results are therefore not generalizable to other chemotherapeutic agents or doses, or to men.

## Conclusions

Extended periods of scalp cooling during EC and paclitaxel chemotherapy for BC seems not improve hair preservation. Shorter durations of scalp cooling might have clear benefits: (i) the intervention would be less time consuming; thus, more patients could be treated per day; and (ii) the intervention might be more comfortable for the patients. Shorter durations may encourage the more widespread use of this intervention.

### Supplementary information

Below is the link to the electronic supplementary material.

Supplementary file1 (PDF 738 KB)

Supplementary file2 (PDF 635 KB)

Supplementary file3 (PDF 504 KB)

Supplementary file4 (PDF 109 KB)

## Data Availability

No datasets were generated or analysed during the current study.
