# Supplementary material for: Effectiveness of increasing the scalp cooling duration to prevent alopecia during adjuvant chemotherapy for breast cancer: a randomized pilot study
Source: Support Care Cancer. 2024 Jun 5;32(7):410. doi: 10.1007/s00520-024-08579-z (PMC11153286; doi:10.1007/s00520-024-08579-z)
Supplement: Supplementary file 1 — Supplementary file1 (PDF 738 KB) [file 520_2024_8579_MOESM1_ESM.pdf]

Effectiveness of increasing the scalp cooling duration to prevent alopecia during adjuvant chemotherapy for breast cancer: a randomized pilot study

Edith Carton[1], Anne Mercier Blas[1], Clément Perret[1], Marcelle Le Bihan[2]

[1] CHP Saint Grégoire, ICRB, Oncologie-Radiothérapie - boulevard de la Boutière, 35760 Saint Grégoire, France

[2] Direction des Soins Territoire Bretagne, Vivalto Santé, 9 boulevard de la Boutière, 35760 Saint Grégoire, France

Corresponding author:

Marcelle Le Bihan, RN, Direction des Soins Territoire Bretagne, Vivalto Santé, 9 boulevard de la Boutière, 35760 Saint-Grégoire, France.

Email: mlebihan@vivalto-sante.com

Online resource 1 : questionnaires

Outcome Evaluation Questionnaire: Patient

Administered after three cycles of epirubicin + cyclophosphamide, after three cycles of paclitaxel, and at the 8-week and 6-month follow-ups.

1 – Your Hair

1. How would you rate your hair loss after the three cycles of chemotherapy?
   ☐ Total loss ☐ Partial loss ☐ No loss *(go directly to question 4)*
2. How would you rate the quality of your hair regrowth (circle the number)?
   No regrowth 0 1 2 3 4 5 6 7 8 9 10 Regrowth of hair as it was prior to treatment
3. What is your level of satisfaction with your hair regrowth (circle the number)?
   Completely dissatisfied 0 1 2 3 4 5 6 7 8 9 10 Completely satisfied
4. One of your friends will have the same chemotherapy treatment for breast cancer. Would you recommend that they wear the cooling cap as you did (circle the number)?
   No, not at all 0 1 2 3 4 5 6 7 8 9 10 Yes, I would try to persuade them
5. Have you had to wear a head covering (toupee or wig, turban, scarf, hat, cap…)? ☐ Yes ☐ No
   If so, at what date? _ _ / _ _ / _ _ _ _
   Any comments:

2 – Other

What is your levels of satisfaction with the regrowth of your (circle the number)

- eyelashes?
  Completely dissatisfied 0 1 2 3 4 5 6 7 8 9 10 Completely satisfied
- eyebrows?
  Completely dissatisfied 0 1 2 3 4 5 6 7 8 9 10 Completely satisfied
- public hair?
  Completely dissatisfied 0 1 2 3 4 5 6 7 8 9 10 Completely satisfied
- leg hair?
  Completely dissatisfied 0 1 2 3 4 5 6 7 8 9 10 Completely satisfied

Any comments:

3 – Your Nails

Did you have any problems with your nails before the chemotherapy? ☐ Yes ☐ No

If so, is this still the case? ☐ Yes ☐ No

Any comments:

Outcome Evaluation Questionnaire: Nurse

Administered after three cycles of epirubicin + cyclophosphamide, after three cycles of paclitaxel, and at the 8-week and 6-month follow-ups.

1. Does the patient have alopecia? ☐ Yes ☐ No
   If so,
   ☐ Grade 1 ☐ Grade 2 ☐ Grade 3 ☐ Grade 4
   Grade 1: <50% loss of hair Grade 2: >50% loss of hair
   Grade 3: total alopecia Grade 4: Persistent alopecia

2. Does the patient wear a head covering (wig, scarf…)?
   ☐ Yes ☐ No
3. Did you have any difficulty putting the cooling cap on?
   ☐ Yes ☐ No
4. Did you have any difficulty taking the cooling cap off?
   ☐ Yes ☐ No

   Any comments:

Tolerability and Satisfaction Questionnaire

Administered at the end of each session of chemotherapy.

1) How would you rate the overall tolerability of wearing the cooling cap? Please circle the number that best corresponds to the tolerability during this session.

Perfectly tolerable 0 1 2 3 4 5 6 7 8 9 10 Intolerable

2) Did you experience any of the following symptoms? If so, please indicate how strong they were.

Discomfort (cold):

- Of the head: ☐ Yes ☐ No
  ☐ None ☐ Minimal ☐ Moderate ☐ Severe
- Of the body: ☐ Yes ☐ No
  ☐ None ☐ Minimal ☐ Moderate ☐ Severe
- Headache: ☐ Yes ☐ No
  ☐ None ☐ Minimal ☐ Moderate ☐ Severe
- Sinusitis: ☐ Yes ☐ No
  ☐ None ☐ Minimal ☐ Moderate ☐ Severe
- Neck pains: ☐ Yes ☐ No
  ☐ None ☐ Minimal ☐ Moderate ☐ Severe
- Nausea: ☐ Yes ☐ No
  ☐ None ☐ Minimal ☐ Moderate ☐ Severe
- Aching: ☐ Yes ☐ No
  ☐ None ☐ Minimal ☐ Moderate ☐ Severe
- Flu-like symptoms: ☐ Yes ☐ No
  ☐ None ☐ Minimal ☐ Moderate ☐ Severe
