# Supplementary material for: Effectiveness of increasing the scalp cooling duration to prevent alopecia during adjuvant chemotherapy for breast cancer: a randomized pilot study
Source: Support Care Cancer. 2024 Jun 5;32(7):410. doi: 10.1007/s00520-024-08579-z (PMC11153286; doi:10.1007/s00520-024-08579-z)

Effectiveness of increasing the scalp cooling duration to prevent alopecia during adjuvant chemotherapy for breast cancer: a randomized pilot study

Edith Carton[1], Anne Mercier Blas[1], Clément Perret[1], Marcelle Le Bihan[2]

[1] CHP Saint Grégoire, ICRB, Oncologie-Radiothérapie - boulevard de la Boutière, 35760 Saint Grégoire, France

[2] Direction des Soins Territoire Bretagne, Vivalto Santé, 9 boulevard de la Boutière, 35760 Saint Grégoire, France

Corresponding author:

Marcelle Le Bihan, RN, Direction des Soins Territoire Bretagne, Vivalto Santé, 9 boulevard de la Boutière, 35760 Saint-Grégoire, France.

Email: mlebihan@vivalto-sante.com

Online resource 2. Percentage of patients with a head covering
The percentage of patients who wore a head covering is shown, as reported by the patients themselves and by the nurses following three cycles of EC, three cycles paclitaxel, and at the 8-week and 6-month follow-ups. The number of patients is shown along the x axis.

GROUP A

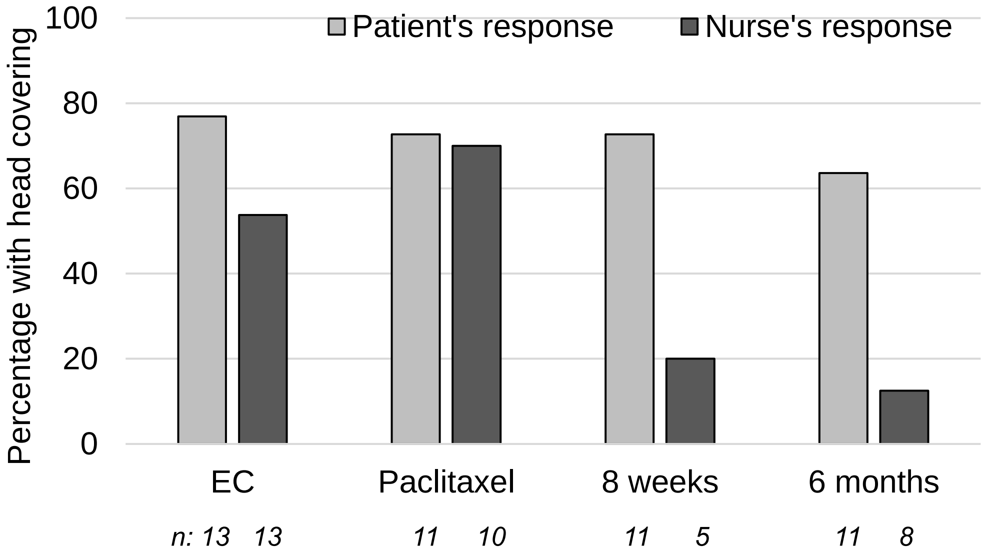

GROUP B

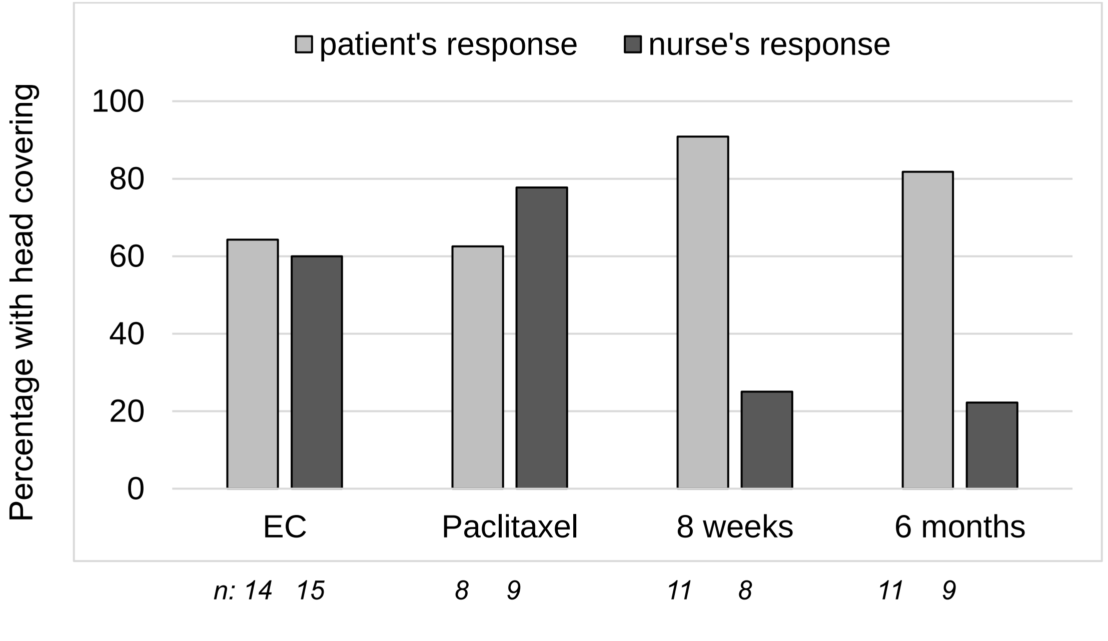

Supplement: Supplementary file 2 — Supplementary file2 (PDF 635 KB) [file 520_2024_8579_MOESM2_ESM.pdf]
