# Supplementary material for: Effectiveness of increasing the scalp cooling duration to prevent alopecia during adjuvant chemotherapy for breast cancer: a randomized pilot study
Source: Support Care Cancer. 2024 Jun 5;32(7):410. doi: 10.1007/s00520-024-08579-z (PMC11153286; doi:10.1007/s00520-024-08579-z)

Effectiveness of increasing the scalp cooling duration to prevent alopecia during adjuvant chemotherapy for breast cancer: a randomized pilot study

Edith Carton[1], Anne Mercier Blas[1], Clément Perret[1], Marcelle Le Bihan[2]

[1] CHP Saint Grégoire, ICRB, Oncologie-Radiothérapie - boulevard de la Boutière, 35760 Saint Grégoire, France

[2] Direction des Soins Territoire Bretagne, Vivalto Santé, 9 boulevard de la Boutière, 35760 Saint Grégoire, France

Corresponding author:

Marcelle Le Bihan, RN, Direction des Soins Territoire Bretagne, Vivalto Santé, 9 boulevard de la Boutière, 35760 Saint-Grégoire, France.

Email: mlebihan@vivalto-sante.com

Online resource 3. Quality of hair regrowth at the 8-week and 6-month follow-ups

The patients rated the quality of their hair regrowth on a scale of 0–10, with zero representing no regrowth and ten representing regrowth of the hair as it was prior to chemotherapy. The plots show the mean values, and the error bars show the standard deviations. The number of patients for each mean is shown along the x axis. The asterisk indicates $p < 0.05$.

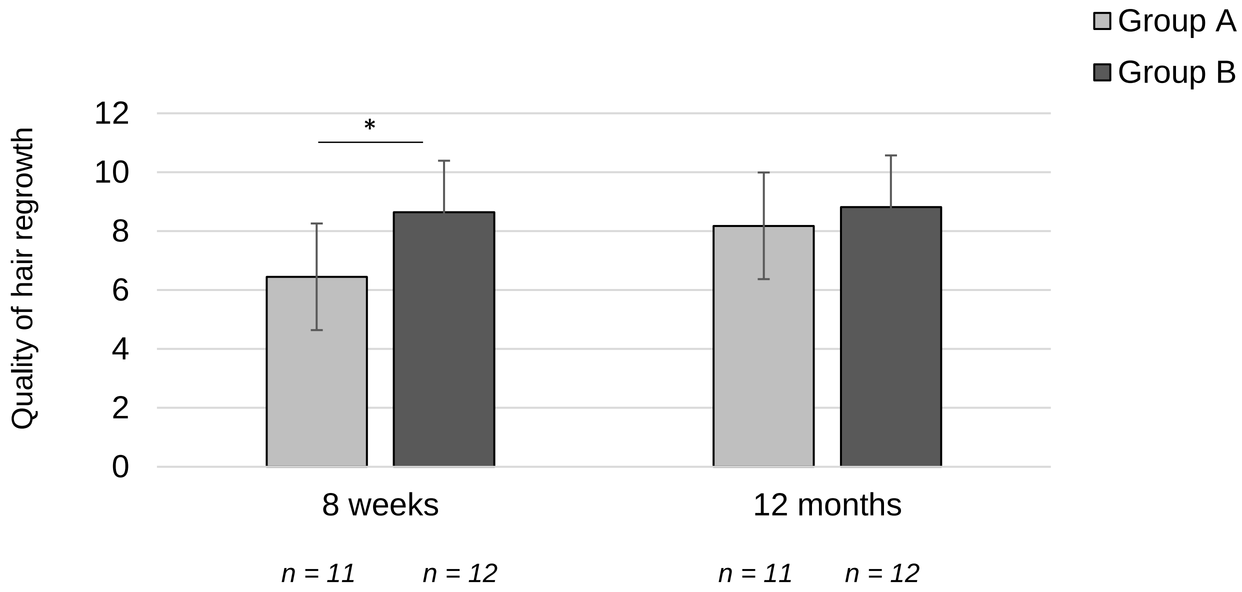

Supplement: Supplementary file 3 — Supplementary file3 (PDF 504 KB) [file 520_2024_8579_MOESM3_ESM.pdf]
