# Supplementary material for: Effectiveness of increasing the scalp cooling duration to prevent alopecia during adjuvant chemotherapy for breast cancer: a randomized pilot study
Source: Support Care Cancer. 2024 Jun 5;32(7):410. doi: 10.1007/s00520-024-08579-z (PMC11153286; doi:10.1007/s00520-024-08579-z)

Effectiveness of increasing the scalp cooling duration to prevent alopecia during adjuvant chemotherapy for breast cancer: a randomized pilot study

Edith Carton[1], Anne Mercier Blas[1], Clément Perret[1], Marcelle Le Bihan[2]

[1] CHP Saint Grégoire, ICRB, Oncologie-Radiothérapie - boulevard de la Boutière, 35760 Saint Grégoire, France

[2] Direction des Soins Territoire Bretagne, Vivalto Santé, 9 boulevard de la Boutière, 35760 Saint Grégoire, France

Corresponding author:

Marcelle Le Bihan, RN, Direction des Soins Territoire Bretagne, Vivalto Santé, 9 boulevard de la Boutière, 35760 Saint-Grégoire, France.

Email: mlebihan@vivalto-sante.com

Online resource 4. Changes in the patients' quality of life over the course of the study
The mean changes in the Global Health Status Scale score from baseline (prior to chemotherapy) are shown for group A and group B following three cycles of EC, three cycles paclitaxel, and at the 8-week and 6-month follow-ups. The number of patients for each mean is shown along the x axis.

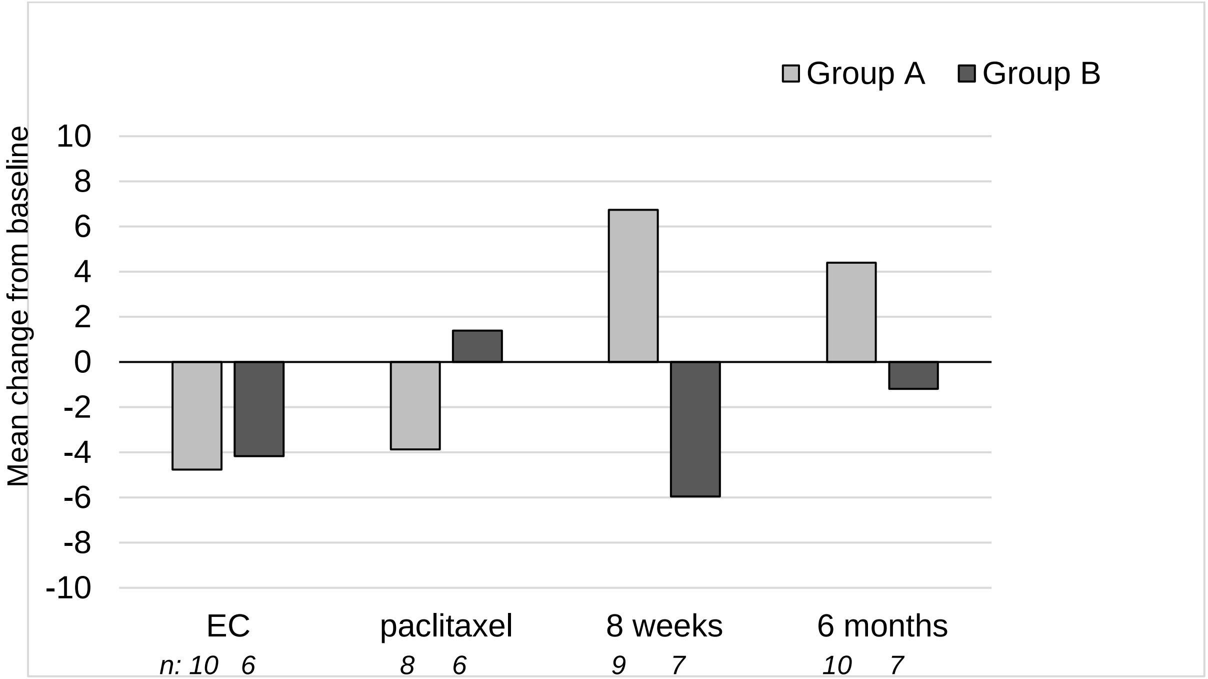

Supplement: Supplementary file 4 — Supplementary file4 (PDF 109 KB) [file 520_2024_8579_MOESM4_ESM.pdf]
